# Butyrate: a bridge between intestinal flora and rheumatoid arthritis

**DOI:** 10.3389/fimmu.2024.1475529

**Published:** 2024-10-16

**Authors:** Yang Cao, Jingjing Chen, Jing Xiao, Yujie Hong, Ke Xu, Yan Zhu

**Affiliations:** ^1^ Second Clinical College, Anhui University of Chinese Medicine, Hefei, Anhui, China; ^2^ School of Sports Health, Shenyang Sport University, Shenyang, Liaoning, China; ^3^ The Second Affiliated Hospital of Anhui University of Traditional Chinese Medicine, Hefei, Anhui, China

**Keywords:** butyrate, intestinal flora, intestinal barrier, bone erosion, immunity, rheumatoid arthritis

## Abstract

In patients with rheumatoid arthritis (RA), intestinal flora imbalance and butyrate metabolism disorders precede clinical arthritis and are associated with the pathogenesis of RA. This imbalance can alter the immunology and intestinal permeability of the intestinal mucosa, leading to damage to the intestinal barrier. In this context, bacteria and their metabolites can enter the bloodstream and reach the distant target tissues of the host, resulting in local inflammation and aggravating arthritis. Additionally, arthritis is also exacerbated by bone destruction and immune tolerance due to disturbed differentiation of osteoclasts and adaptive immune cells. Of note, butyrate is a metabolite of intestinal flora, which not only locally inhibits intestinal immunity and targets zonulin and tight junction proteins to alleviate intestinal barrier-mediated arthritis but also inhibits osteoclasts and autoantibodies and balances the immune responses of T and B lymphocytes throughout the body to repress bone erosion and inflammation. Therefore, butyrate is a key intermediate linking intestinal flora to the host. As a result, restoring the butyrate-producing capacity of intestinal flora and using exogenous butyrate are potential therapeutic strategies for RA in the future.

## 1 Introduction

Rheumatoid arthritis (RA) is a chronic autoimmune disease characterized by chronic inflammation and cartilage and bone destruction (1). Currently, RA is considered to develop as a result of a combination of genetic and environmental factors (2). Moreover, intestinal flora imbalance is an important environmental factor present before and throughout the pathogenesis of RA (3, 4). Intestinal flora imbalance is one of the primary factors triggering and driving abnormal systemic immunity in RA since changes in the relative abundance of different bacterial strains may alter the immune profile in the host, affecting inflammation in RA (5). For example, Prevotella copri and Lactobacillus salivarius can stimulate immune response and worsen the severity of arthritis. However, some other strains such as Haemophilus spp and Faecalibacterium prausnitzii have been shown to be negatively correlated with immune response and may benefit RA patients from immune modulation (6, 7). More importantly, intestinal flora imbalance can induce changes in metabolites, such as a decrease in butyrate, and there is a correlation between the two (8, 9).

**Figure 1:**
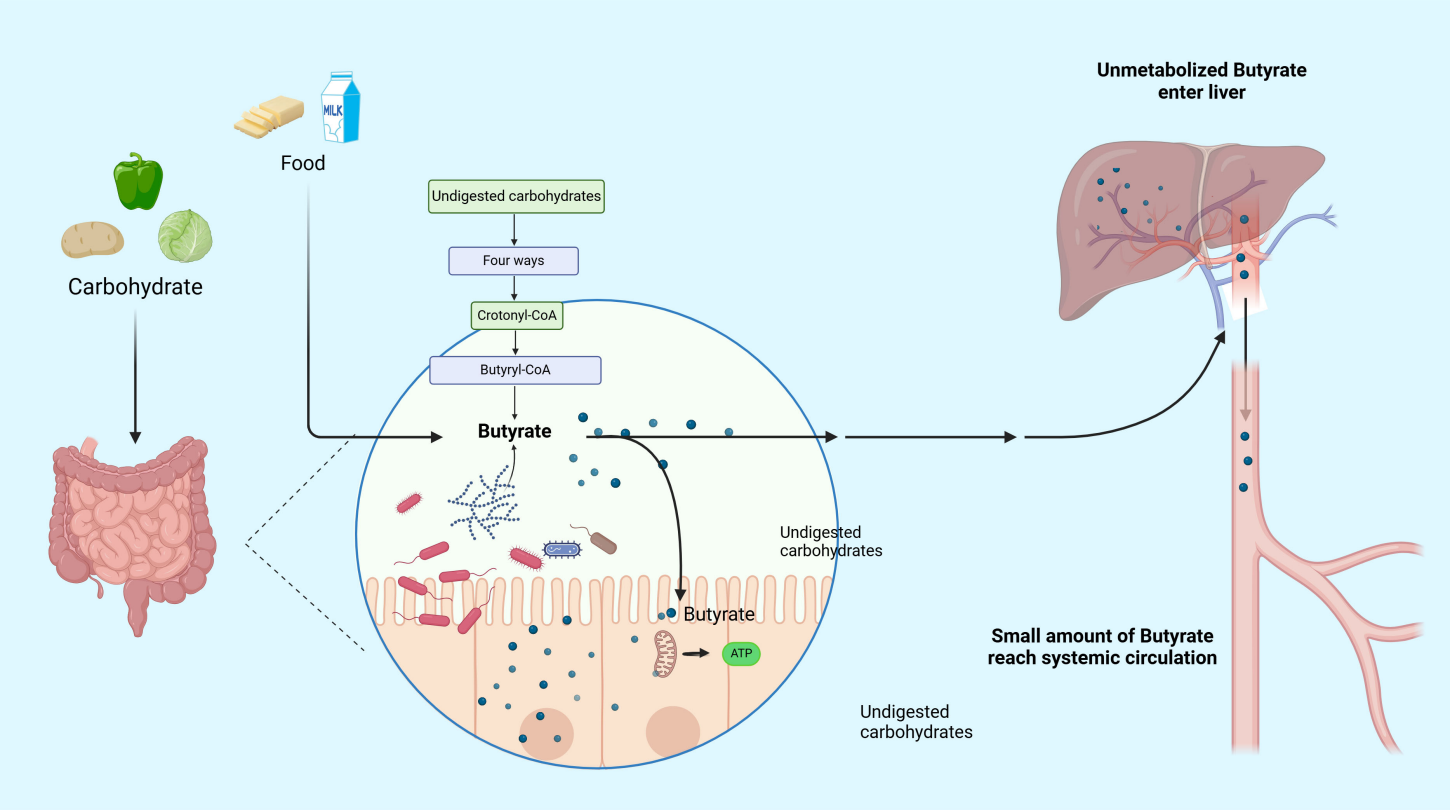
Metabolic pathway of butyrate. Butyrate can be obtained in small exogenous quantities from certain foods. However, the majority of butyrate in the human body is produced through the glycolysis of undigested host carbohydrates by the gut microbiota. Most of the butyrate produced is absorbed and utilized by the colonic epithelium, which serves as the main energy source for the intestinal epithelium. A small proportion is absorbed by the liver, and the remaining extremely low concentration of butyrate enters the circulation for metabolism.

As a short-chain fatty acid, exogenous butyrate can be obtained in small amounts from some foods, such as ruminant milk, butter, and yogurt (10). In the human body, most endogenous butyrate is derived from different short-chain fatty acid molecules (mainly acetic, propionic, and butyric acids) produced due to the glycolysis of undigested carbohydrates, such as cellulose, xylan, and pectin by the intestinal microbiota, particularly colonic bacteria (11). There are four predominant pathways for endogenous butyrate production, namely the acetyl-coenzyme A (CoA), glutarate, 4-aminobutyrate, and lysine pathways, all of which merge at the central step of conversion of crotonyl-CoA to butyryl-CoA which is eventually converted to butyrate by various butyryl-CoA transferases (12). Among bacteria, butyrate-producing bacteria have high abundances in adults (> 20% of total bacterial community), and major members of Lachnospiraceae and Ruminococcaceae (members of the phylum Bacillota) are butyrate producers (12, 13). Produced butyrate is mostly absorbed and utilized by the colonic epithelium, which is the main energy source for the intestinal epithelium, and subsequently is absorbed in a small portion by the liver. Additionally, the remaining butyrate (extremely low concentration) enters the circulation to be metabolized (14, 15). Recent studies have unraveled that butyrate production and consumption are imbalanced in RA and that the immune status and joint symptoms of RA model rats can be improved by dietary butyrate supplementation (16, 17). Another study has elucidated that the ingestion of resistant starch, inulin, and apple cider vinegar can increase butyrate production in the colon of the subject population and subsequently affect systemic immune response (18). Hence, these observations support that butyrate has great potential for the treatment of RA. However, the specific mechanism of action has not yet been fully understood. Therefore, this review tried to investigate the underlying cellular mechanism of butyrate in RA and elaborate the effects of butyrate on RA, aiming to provide ideas for new research and treatment for RA (**Figure 1**).

## 2 Butyrate ameliorates intestinal barrier-mediated arthritis

The gut-joint axis has become a research hotspot. Although it has been reported that intestinal dysbacteriosis is unlikely to trigger the onset of RA as a secondary phenomenon (19), more reports subscribe to the “mucosal origin hypothesis” where intestinal flora imbalance compromises the mucosal barrier, which allows for the entry of bacteria and their metabolites into the host bloodstream to reach distant target tissues, thereby causing localized inflammation (20). For example, the outer membrane vesicles of Fusobacterium nucleatum, a highly expressed intestinal microbe in the host, can elicit localized inflammation by acting on synovial macrophages via the Rab5a-YB-1 axis, therefore aggravating the symptoms of collagen-induced arthritis (CIA) in mice (21). Additionally, it has been shown that peptidoglycan is found in the synovial tissue of RA patients, which is derived from the mucosa, which suggests that mucosal lesions may lead to inflammation in the joint microenvironment (22). Overall, the altered composition of the intestinal mucosa is an inducer for aberrant immunity in distal target tissues of RA patients. Furthermore, arthritis-induced intestinal flora dysbiosis can be partially restored to baseline characteristics after treatment, and restoration of microbial abundance and butyrate metabolism contributes to immune and intestinal homeostasis (23). However, little is known about the specific mechanism of gut microbes causing the immune response in arthritis and more mechanistic studies are needed for validation in the future. Maintaining gut barrier function to benefit RA patients is a therapeutic pathway.

### 2.1 Butyrate maintains intestinal barrier function by restraining intestinal immune responses in RA

The intestinal mucosal immune barrier coordinates with immune responses via local immunity, which eliminates invading pathogens in the physiological state to protect the organism and prevents the over-activation of systemic immune responses, such as the secretion of immunoglobulin A (IgA) from plasma cells (24). When damaged or abnormal, the intestinal immune barrier loses its ability to eliminate pathogens, which disrupts intestinal homeostasis, leading to unrestricted activation of immune responses and the development of diseases (25). Intestinal mucosal immunity is also one of the hypotheses for RA pathogenesis, which suggests that arthritis results from interactions between the mucosal immune system and an abnormal local microbiota (26).

It has been widely recognized that intestinal ecological imbalance induces intestinal mucosal immunity, triggering the development of RA. However, there are different experimental results on the inflammation that result in intestinal mucosal inflammation in RA except for T helper 17 (Th17) cells (**Table 1**) (4, 15, 27), which may be related to different experimental sampling sites. For instance, the microbiota in the ileum may be more representative of CIA-induced inflammation than that in the cecum, and the mRNA expression of the inflammatory factors interleukin (IL)-1β and IL-17A is significantly elevated only in the epithelium of the ileum and the lamina propria of the mucosal layer (28). However, there is no denying that these results indicate the presence of a pro-inflammatory immune response in the intestinal mucosa and influence the progression of RA. In addition, intestinal dysbacteriosis and Th17-mediated intestinal mucosal immunity are present during the development of RA and precede clinical joint symptoms (4). A prior study also unveiled that intestinal dysbacteriosis and Th17-mediated intestinal mucosal immunity, as well as their characteristic cytokines IL-17A and IL-22, were also observed in CIA mice before the occurrence of arthritis and that antibiotic interventions alleviated arthritis modulated by intestinal ecology and mucosal immune imbalance (27). Accordingly, alterations in the intestinal microbiota and inflammation precede joint symptoms, highlighting that modulating the intestinal inflammatory state may be a potential strategy for the prevention and treatment of RA. Previous research revealed that the use of α-glucosidase inhibitors alleviated the imbalance in the ratio of Th17 to regulatory T (Treg) cells (immune cells in the intestinal mucosa) due to intestinal flora imbalance and reduced the arthritic symptoms of CIA mice (29). Consequently, restricting intestinal mucosal immunity and maintaining intestinal barrier intactness emerge as one of the intervention approaches for RA.

**Table 1:** Gut barrier dysfunction in RA.

Type	Subjects	Material	UP		Unchanged	Down	References
			Immune cells	cytokines			
Immune barrier	CIA mice	colon	Th17	IL-17A, IL-22, IL-23	IFN-γ^*^, IL-12p70, IL-1β,IL-6, IL-10,TNF-α		(27)
Immune barrier	FMT^*^+CIA mice	intestine	Th17	IL-17			(4)
Immune barrier	FMT mice	intestine	Th17	IL-17A, IL-22	Th1,Th2		(4)
Immune barrier	CIA mice	intestine, colon	Th1,Th17, Th2 (late stage) Treg (late stage)				(16)
Permeability barrier	RA patients	colon			claudin-2, occludin	ZO-1	(42)
Permeability barrier	Patients with active RA	Ileum or serum	Zonulin			occludin claudin-1	(16)
Permeability barrier	CIA mice	intestine, colon	Zonulin, claudin-2, claudin-15			ZO-1, occludin	(16)
Permeability barrier	Intestinal epithelium Cells		Zonulin				(16)

As has been confirmed by *in vitro* experiments on human intestinal epithelial cells, in the case of an inflammatory response in the intestine, butyrate within the physiological concentration range (0.0625-0.5 mM) would attenuate CD4 T cell activation in the lamina propria of the human intestine, the proliferation of Th1, Th17, and Th22 (autoreactive T cells), and cytokine IL-17 production. These effects may be tightly implicated in the ability of butyrate to enhance histone acetylation (30). Histone deacetylases (HDACs) are enzymes that remove acetyl groups from histones and can serve as key regulators of gene expression (31), Butyrate functions as an inhibitor of HDACs and plays a positive role in anti-inflammation and barrier protection (32). Butyrate is able to completely prevent intestinal epithelial permeability induced by cytokines such as IL-1β, tumor necrosis factor-α (TNF-α), and IL-17a for a long time (33). Furthermore, a former study displayed that the level of HDAC-3 and the proportion of Th17 (inflammatory cells) were reduced and colonic mucosal integrity was protected in butyrate-treated mice (34). These findings notably indicate that HDACs are the action mechanism of butyrate in intestinal mucosal inflammation; adequate levels of butyrate can restrict immunity and maintain intestinal homeostasis. Nevertheless, the specific mechanism of butyrate in enhancing histone acetylation remains unknown. Some researchers have proposed that there are butyrate response elements in the promoters of butyrate-responsive genes. These elements can recruit HDACs to the promoter by inhibiting transcription factors specificity protein 1/specificity protein 3 (Sp1/Sp3), thereby resulting in excessive histone acetylation (35). Additionally, another cellular experiment has demonstrated that butyrate enhances the accessibility of hypoxia-inducible factor 1α to the IL22 promoter binding site in CD4 T cells via histone acetylation, resulting in IL-22 production and thereby exerting a protective effect on the intestinal mucosal barrier (36). Therefore, the mechanism of butyrate and HDACs is closely related to the promoter. However, in-depth exploration of the specific mechanism is still needed. In the future, the resolution of these issues will help to further understand the mechanism of butyrate in benefiting arthritis via protecting the intestinal mucosal barrier (**Table 1**).

### 2.2 Butyrate maintains intestinal barrier health by constraining intestinal permeability

The intestinal epithelial barrier not only regulates the transport of macromolecules between the environment and the host but also isolates the internal and external environments by repressing the infiltration of external antigens and the leakage of endogenous substances, thus contributing to intestinal homeostasis maintenance. This function of the intestinal epithelial barrier relies on intercellular junctions, particularly tight junctions (TJs) (37, 38). Consequently, intestinal barrier dysfunction is associated with altered expression of epithelial TJs molecules. TJs have the core function of forming a paracellular permeability barrier, are composed of transmembrane proteins including occludin and claudins, and are connected to the cytoskeleton via zonula occluden (ZO) (39). The claudin family of proteins has diverse functions. In terms of endothelial barrier maintenance, claudins are a critical component of TJs, which can be categorized into barrier-forming (such as claudin-5) and channel-forming (such as claudin-2) proteins that regulate the opening and closing of endothelial TJs (40, 41). As reported, TJs proteins are altered and serum biomarkers of intestinal permeability are upregulated in the colon of RA patients, and impaired intestinal integrity is associated with inflammation in RA (42). On the contrary, an earlier study showed that the use of total methanol extract of jasmine, a TJs protein protector, can preserve TJs in intestinal epithelial cells, which preserved TJs in intestinal epithelial cells and ameliorated bone and articular cartilage damage and synovial and periarticular tissue inflammation in arthritic rats (43). Conclusively, increased intestinal permeability due to changes in TJs is directly associated with the development of arthritis, and enhanced TJs polymerization may represent relief of RA symptoms. In addition, zonulin is by far the only physiological regulator of intercellular TJs described, it can interact with specific intestinal epithelial surface receptors and subsequently regulate the permeability of TJs (44, 45). Therefore, targeting zonulin or TJs can prevent damage to the intestinal mucosal barrier function. Tajik et al. observed that butyrate treatment not only decreased serum zonulin concentrations but also restored intestinal barrier function by modulating the mRNA expression of TJs proteins, including claudin-2, thereby improving arthritis (16), illustrating that butyrate improves intestinal barrier function and attenuates RA by mediating TJ proteins via zonulin.

**Figure 2:**
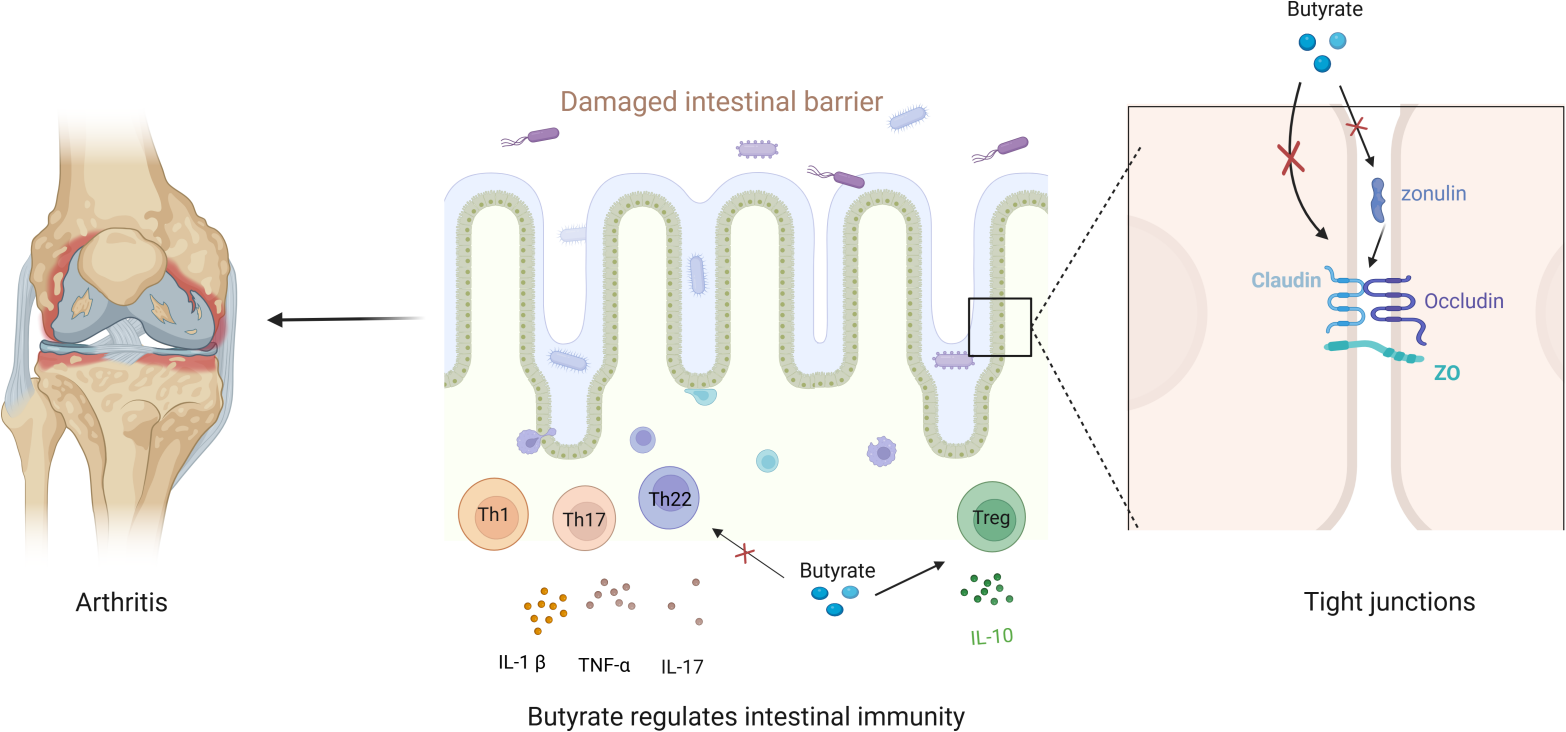
Butyrate maintains gut barrier health to alleviate arthritis. Disruption of the gut barrier aggravates arthritis, and butyrate enhances both the immune barrier and the permeability barrier. Regarding the immune barrier, butyrate restores the balance between pro-inflammatory and anti-inflammatory factors to maintain a healthy intestinal immune barrier. With respect to the permeability barrier, butyrate regulates TJ or targeted zonulin to limit intestinal permeability and maintain a healthy intestinal barrier.

Taken together, when the intestinal barrier is disrupted, harmful substances in the intestine (such as Prevotella copri and the outer membrane vesicles of Fusobacterium nucleatum) can pass through the intestinal barrier and act on the distant target organs of RA (6, 21). Therefore, the damaged intestinal barrier is a crucial factor in RA development, and restoring damaged intestinal barrier function may be the entry point for treating RA. The role of butyrate in maintaining intestinal barrier health involves restoring immune function and enhancing the function of TJs. On the one hand, butyrate can limit the immune response in the gut without over-activating it, thus protecting intestinal barrier health. On the other hand, butyrate can regulate the permeability of the intestinal barrier through opening and closing the TJs (a significant link between the epithelial cells of the intestinal mucosa) to exert protective effects. Therefore, butyrate is a potential therapeutic target for RA with its role in maintaining gut health (**Figure 2**).

## 3 Butyrate ameliorates osteoporosis and bone erosion

Osteoporosis and bone erosion can severely jeopardize the quality of life of RA patients and are major prognostic challenges for RA. Moreover, treatment with glucocorticoids has greater negative impacts on bone health of RA patients (46). Therefore, it is necessary to develop measures for preventing bone loss. The following section describes the role of butyrate in improving bone quality in terms of osteoclast-involved bone metabolism and autoantibody-mediated bone erosion.

### 3.1 Butyrate depresses osteoclastogenesis via receptor activator of nuclear factor-κB ligand and HDAC2 pathways

In the physiological state, normal bone metabolism is maintained through dynamic interactions between osteoblasts and osteoclasts (OCs). In the disease state, such as RA, OCs differentiate and increase, leading to focal articular bone destruction and generalized bone loss (47). OCs that regulate bone metabolism originate from the monocyte/macrophage lineage of bone-marrow hematopoietic stem and progenitor cells, and OC precursors are driven by both macrophage colony-stimulating factors and RANKL to differentiate into terminal OCs (48). OCs are the only bone-resorbing cells in the body and also the only cells secreting tartrate-resistant acid phosphatase (TRAP) (49). Therefore, inhibiting osteoclastogenesis and OC differentiation to balance bone metabolism is a strategy to sustain bone homeostasis in RA. A prior study demonstrated that TNF-α directly affected RANKL expression in osteoblasts and increased OC production (50). Another study showed that peripheral blood mononuclear cells from RA patients had higher potential for TNF-induced OC differentiation than those from healthy donors (51). Mechanistically, RANKL is an inducer of OC differentiation and activation, which can recruit articulating proteins such as tumor necrosis factor receptor-associated factor 6 (TRAF6) by binding to RANK (52). TRAF6 possesses a specific binding site to the cytoplasmic structural domain of RANK and activates the major transcription factor Nuclear Factor Of Activated T Cells 1 (NFATc1) in the downstream pathway, thereby orchestrating OC differentiation (53, 54). Furthermore, RANKL-activated OC differentiation can be suppressed by negatively regulating TRAF6 and inhibiting the transcriptional and translational expression of NFATc1 (55, 56). As a result, osteoclastogenesis and bone loss can be prevented by studying the RANKL-induced pathway to block TRAF6 and NFATc1 activities and OC precursor differentiation. Notably, Lucas et al. found that butyrate significantly downregulated TRAF6 and NFATc1, two essential OC signaling components, at early time points after RANKL stimulation, thereby ameliorating bone destruction, evidenced by an increase in whole-body bone mass and a reduction in the number of OCs in the affected paws and in the serum level of the bone resorption marker CTX-I in arthritic mice (57), which is a crucial mechanism for combating bone loss.

HDAC2 is a key positive regulator of osteoclastogenesis and bone resorption, which favors OC formation when overexpressed and reduces osteoclastogenesis and ameliorates bone destruction when repressed (58). Earlier research displayed that similar to HDAC2 inhibitors, butyrate suppressed osteoclastogenesis and markedly lowered the levels of osteoclastogenic markers, such as TRAP, integrin β3, matrix metalloproteinases 9, and TNF-α in CIA mice (59). To sum up, OCs are major players in joint and bone destruction, and it is urgent to develop more effective therapeutic approaches for abolishing the destructive nature of OCs in the pathological state. Moreover, the therapy that butyrate targets OCs by downregulating RANKL and HDAC2 may be highly beneficial for the treatment of both RA-related bone destruction and osteoporosis. Although the efficacy of butyrate needs to be further investigated, butyrate may be an emerging therapy for RA patients.

### 3.2 Butyrate relieves bone erosion by decreasing autoantibody production

Autoantibodies [such as anti-citrullinated peptide antibodies (ACPA)] are mainly secreted and produced by autoreactive B cells after they differentiate into long-lived plasma cells and participate in humoral immunity (60). The European League Against Rheumatism/American College of Rheumatology has put forward the classification criteria for RA in 2010, including ACPA (61). As discovered by pathological mechanisms in recent clinical research, as a serum reactant, ACPA has a direct connection with bone erosion in RA patients, with the manifestations of increased possibility of severe cartilage and bone damage, and a worse prognosis accompanied by severe joint deformities (62, 63). Mouse studies have reported that ACPA can trigger OC activation and bone resorption in bone marrow (64). Accordingly, the presence of autoantibodies not only signifies the onset of autoimmunity but also aids in diagnosis and is associated with prognosis, and these immune responses may be attenuated by reducing the impact of autoantibodies. In RA, butyrate-producing and butyrate-depleting bacteria are correlated with ACPA and bone destruction, and butyrate-associated intestinal microbes and butyrate concentrations are substantially negatively correlated with autoantibody titers in RA. Additionally, dietary butyrate confers benefits for mouse models of RA by reducing antibody production (17). However, the current experimental results are insufficient to explain the underlying specific mechanism of action. The mechanism of butyrate or butyrate-related bacteria in autoantibodies should be further investigated in future research, which will help to better update the potential therapeutic targets for RA treatment.

**Figure 3:**
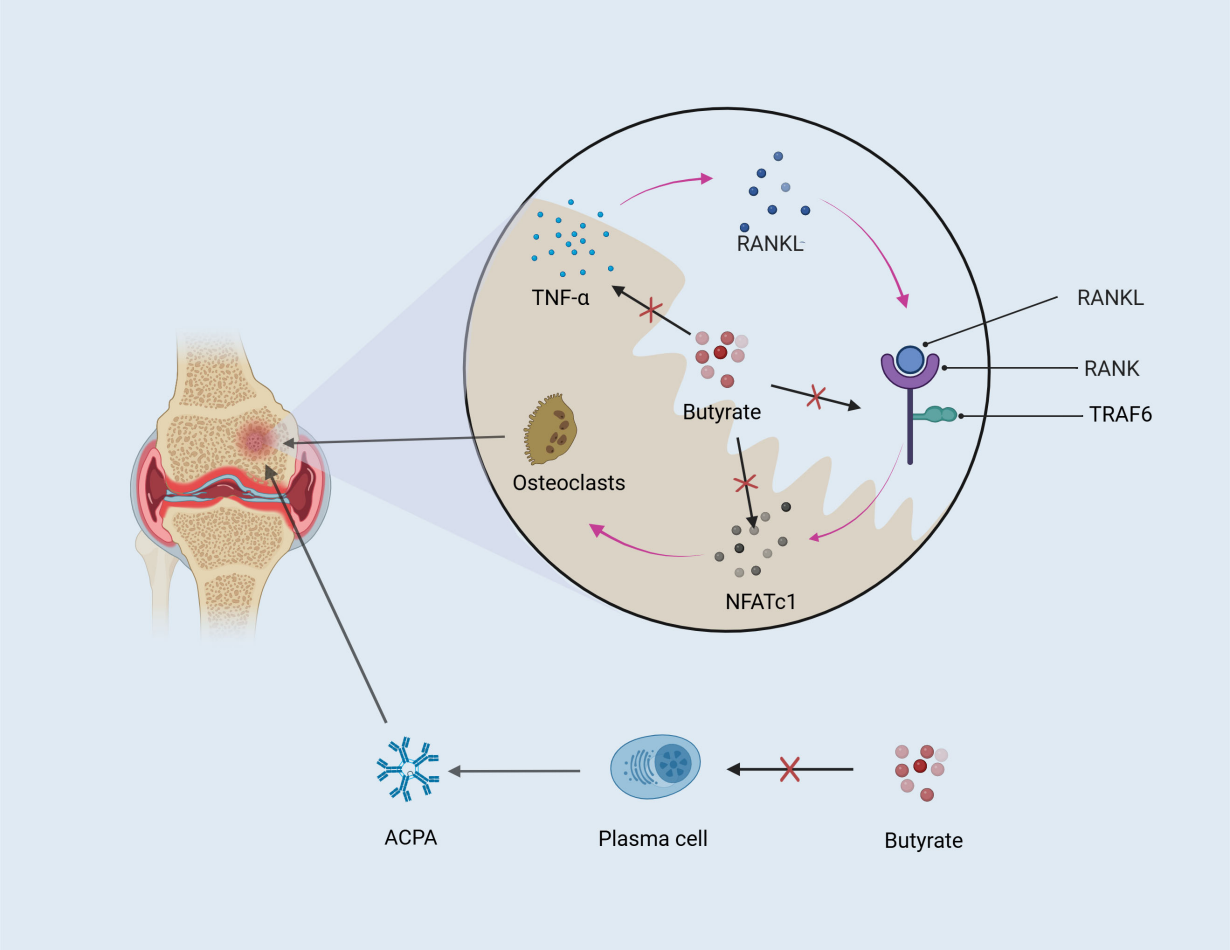
Butyrate ameliorates bone loss. OC and autoantibodies both contribute to bone erosion. Butyrate mitigates bone erosion by inhibiting TNF-α and the RANKL downstream factors TRAF6 and NFATc1, which regulate OC differentiation. Additionally, autoantibodies produced by plasma cells differentiated from B cells are positively correlated with bone destruction in RA, and butyrate may alleviate bone loss by reducing autoantibody production.

In conclusion, OC activation and the increase of ACPA are two important factors for the imbalance of bone homeostasis in RA. Butyrate inhibiting osteoclast activation is related to HDACs and RANKL pathways. In terms of improving ACPA-caused bone erosion, the positive effect of butyrate on autoantibodies has been clarified (17), even though there has not been published research pointing out the specific mechanism of butyrate (**Figure 3**).

## 4 Butyrate inhibits chronic inflammation by modulating adaptive immune cells

Adaptive immunity is a powerful host defense tool, which is classically dominated by lymphocytes of T- and B-cell lineages. The immune system strictly controls the differentiation and development of T and B lymphocytes, ensuring its reactivity to pathogens and foreign antigens while increasing tolerance to itself through cellular immune effects. The infiltration of self-reactive T and B lymphocytes has long been recognized as the main contributor to immune tolerance in RA. Recent reports have confirmed that in RA, the conversion of potentially protective T cells to pathogenic T cells precedes the onset of clinical inflammation (65), which provides a novel promising therapeutic intervention for RA. B cell-related genes may also be a potential target for the clinical diagnosis and immunotherapy of RA (66). Therefore, further research on the cellular immune progression of RA greatly advances the understanding of the mechanisms of chronic inflammation and provides additional theoretical support for the therapeutic mechanism of butyrate.

### 4.1 Butyrate limits immune responses by mediating Th17/Treg balance

In T cell-mediated immune responses, primary CD4 T cells differentiate into pro-inflammatory Th17 cells and anti-inflammatory Treg cells. Treg cells negatively affect inflammation by releasing the anti-inflammatory cytokines IL-10 and transforming growth factor (TGF)-β (67). When activated, effector Th17 cells secrete IL-17, IL-6, and TNF-α to boost chronic immunity-mediated inflammation (68). Th17/Treg homeostasis is influenced by impairing the function of Treg cells, lowering the number of Treg cells, and enhancing the number of Th17 cells (69). After pharmacologic treatment, the ratio of Th17/Treg can rebalance, thus yielding clinical remission (70), indicating that Th17/Treg balance is affected by increases in cellularity. Therefore, the relationship between Th17/Treg imbalance and the production of pro- and anti-inflammatory factors is essential for the development of autoimmunity, chronic inflammation, and damage in the joints of RA patients.

CD4+CD25+ Treg cells expressing forkhead box protein p3 (Foxp3) are responsible for maintaining immune self-tolerance and immune homeostasis, which modulate the transcription of Foxp3 in the presence of the enhancer conserved non-coding sequence 1 (CNS1) to determine the differentiation of the Treg profile, hence preventing autoimmunity and maintaining immune homeostasis through amplification (71). Functionally, the constitutively high expression of the immune checkpoint receptor cytotoxic T-lymphocyte-associated antigen-4 (CTLA-4) can dependently decrease the T-cell stimulatory activity of antigen-presenting cells, and the specific deficiency of CTLA-4 can lead to severe systemic autoimmunity (72). Th17 cells are another class of pro-inflammatory cells involved in immunity, in which HDAC8 is highly expressed to act as a deacetylase for estrogen-related receptor alpha (ERRα), enhancing the transcriptional activity of ERRα (59, 73). In addition, the specific inhibition of nuclear receptor subfamily 1 group D member 1 (NR1D1), a Th17 differentiation-related gene, delays the development of CIA by maintaining Treg populations but decreasing Th17 cell differentiation (74). All of these mechanisms are important for sustaining Th17/Treg homeostasis.

Butyrate can exert specific regulatory effects on the T cell lineage. A study revealed that butyrate produced by intestinal microbes during starch fermentation fosters extra-thymic Treg cell differentiation in a CNS1-dependent manner, ultimately influencing the balance between pro- and anti-inflammatory mechanisms (75). Another study displayed that butyrate elevated the number of Treg cells expressing CTLA4 and Foxp3 and modulated HDAC8 in CD4 T cells in a dose-dependent safe manner. This study also demonstrated that butyrate controlled the transcriptional activity of ERRα and NR1D1 and reduced the number of Th17 cells and the expression of Th17 cell-related pro-inflammatory cytokines IL-17, IL-1β, IL-6, and TNF-α in CIA mice, ultimately lowering the Th17/Treg ratio (59). Intriguingly, the effects of butyrate on Th17 and Treg cells are not balanced. Specifically, a prior study demonstrated that butyrate dose-dependently lowered the expression of Th17 differentiation-related Retinoic Acid Receptor-Related Orphan Receptor γt and IL-17 and increased Foxp3 expression, with more enhancing effects on Treg cells than on inflammatory Th17 cells (76). Hence, butyrate stimulates the differentiation of naïve T cells to Treg and depresses their differentiation into Th17 cells, thereby orchestrating the balance between cellular immunity-mediated anti-inflammatory and pro-inflammatory cytokines, reaffirming butyrate as a potential therapy for RA.

### 4.2 Butyrate modulates T follicular regulatory cell/T follicular helper cell balance to restrict immune responses

Tfh and Tfr cells are terminally differentiated cells discovered in germinal centers (GCs). Specifically, Tfr cells are a specific subpopulation of Treg cells and are identified as CD4+CXCR5+PD-1+Bcl6+Foxp3+CD25- (77). Tfh is another type of GC-resident cells, which facilitate antibody production by increasing GC formation and response (78). Tfh and Tfr cells have opposite functions, among which Tfr cells sustain immune homeostasis by inhibiting antibody production via Tfh-assisted B cells (79), whilst Tfr/Tfh imbalance and Tfr cell-repressed antibody production is required for maintaining immune tolerance. Peripheral blood Tfr/Tfh imbalance appears in RA and new-onset RA patients, and the intestinal flora imbalance-mediated reduction in the number of circulating Tfr cells is negatively correlated with autoantibody levels and RA severity (80, 81). Prior research showed that Tfr/Tfh imbalance changed after treatment in model mice of RA, accompanied by decreased autoantibody levels and increased Tfr cells (82). The same results were also observed in RA patients in stable remission (83), underlining that restoration of immune tolerance by targeting Tfr cells has significant therapeutic potential for RA. Overall, the pathogenesis of RA is associated with immune tolerance impairment and antibody overproduction induced by aberrant Tfr cells.

Butyrate not only reduces Tfh cells (17) but also regulates Tfr/Tfh imbalance by inhibiting the differentiation of HDAC-induced functional Tfr cells, alleviating GC response-modulated swelling of colon-associated lymphoid tissues prior to the onset of CIA and ultimately controlling the development of autoimmune arthritis (84). As a consequence, microbiota-derived butyrate, as a bridge between the intestinal environment and RA patients, can orchestrate autoantibody production in systemic lymphoid tissues by restoring Tfr/Tfh balance, therefore ameliorating RA.

### 4.3 Butyrate modulates B cells and their receptors to affect immune responses

In autoimmune responses, immunity is usually persistently activated by the insufficient number and function of suppressor cells in the circulation and at the inflammatory site, and anti-inflammatory responses are disadvantaged by the reduced number of suppressor cells, such as regulatory B cells (Breg), or by impaired cytokine control of inflammation abatement (85). Breg cells are a group of B cells with the function of suppressing immune responses, which exert functionally inhibitory effects during the inflammatory phase of autoimmunity. An earlier study reported that the arthritis symptoms of mice were aggravated in the absence of Breg cells (86), illustrating that Breg cells are activated in response to inflammatory signals that drive autoimmune diseases, thereby preventing destructive inflammation that would otherwise develop. This effect of Breg cells is related to the fact that Breg cells suppress various immunopathologies, including but not limited to autoimmune diseases, through the release of representative cytokines including IL-10, IL-35, and TGF-β, thereby impeding ongoing immune responses and reestablishing immune homeostasis (87, 88). Conclusively, Breg cells may be potential therapeutic targets for a wide range of immunity-mediated inflammatory conditions.

Breg cells are involved in the immune regulation of RA. Previous research revealed that Breg cells associated with Disease Activity Score 28 and inflammatory markers erythrocyte sedimentation rate and C-reactive protein were reduced in RA patients, which was nullified by conventional synthetic disease-modifying antirheumatic drugs (89). Another study demonstrated that the anti-inflammatory effects of Breg cells in RA might be achieved by modulating the IL-10-producing transcription factor aryl hydrocarbon receptor (AhR), which was highly expressed in CD19+CD21+CD24+ Breg cells and improved the expression of inflammatory factors by regulating Breg cell differentiation and function. In this study, Breg cell differentiation favored pro-inflammatory response and reduced IL-10 expression, thus exacerbating arthritis in AhR-deficient mice (90). Conclusively, as a key regulator of immune tolerance, AhR responds to inflammatory signals and plays a pivotal role in the homeostasis maintenance of Breg cell function as a molecular brake, preventing the differentiation and pro-inflammatory mediator production of Breg cells, illustrating AhR as a potential target for regulating autoimmunity.

There is a partial consensus on the effector function of Breg cells and many similarities in the phenotypes and effector functions of multiple Breg cell subpopulations. Nevertheless, their phenotypes remain controversial (91), which may explain the conflicting effects of butyrate on Breg cells. Additionally, a former study demonstrated that intestinal microbiota-derived butyrate activated AhR, a functional transcriptional marker of CD19+CD21+CD24+ Breg cells, in a Breg cell-dependent manner, which inhibited arthritis and reduced arthritis severity (92). However, another study elaborated that butyrate repressed inflammation in CIA by regulating B cell differentiation via free fatty acid receptor 2 (FFA2), which was abrogated after FFA2 receptor knockdown (93). Hence, butyrate supplementation may be a viable therapy for systemic autoimmune diseases. However, it remains a critical issue to exactly identify the phenotype of the Breg lymphocyte subpopulation and determine the specific mechanism of action of butyrate action (**Table 2**).

**Table 2:** Gut barrier dysfunction in RA.

Immune cells	Medium	Acting point	References
Treg	IL-10, TGF-β	CNS1, CTLA4, Foxp3	(67, 75)
TH17	IL-17, IL-1β, IL-6, TNF-α	HDAC8, ERRα, NR1D1, RORγt	(59, 76)
Tfr/Tfh	Autoantibodies	HDAC	(17)
Bregs	IL-10	AhR, FFA2	(92, 93)

Conclusively, the adaptive immune cells T and B lymphocytes are in a state of immune disorder during inflammation, and the differentiation, development, and secreted cytokines of these cells are implicated in chronic inflammation during RA. Moreover, butyrate can restore immune self-stabilization, favor Th17/Treg and Tfr/Tfh homeostasis, and expand anti-inflammatory Breg cells, ultimately balancing pro-inflammatory and anti-inflammatory responses. This review contributes to the in-depth understanding of the molecular mechanisms of butyrate in the treatment of RA.

## 5 Conclusion and discussion

RA is a multifactorial disease characterized by chronic synovial inflammation and bone destruction. As a critical part of RA, intestinal barrier damage caused by intestinal ecological imbalance can cause and exacerbate RA, which further supports the intestine-joint axis theory. Therefore, targeting intestinal flora translocation or intestinal barrier at the preclinical or clinical stage may be a major breakthrough. The function of butyrate to restore intestinal bacterial homeostasis and intestinal barrier function has been observed in mouse models. Nonetheless, further studies are warranted to explore the role of butyrate in improving RA through intestinal barrier health and the involved specific pathways since studies of butyrate predominately focused on inflammatory bowel disease. In terms of autoantibodies, the function of butyrate in reducing autoantibodies and bone destruction is supported by preliminary data. However, the related mechanism remains incompletely investigated, and many questions, such as the specific binding epitopes of butyrate to ACPA or the mechanism by which butyrate modulates ACPA to ameliorate bone erosion, have not yet been addressed, which calls for additional studies to examine the epitopes, characterization, and effector functions of these autoantibodies. In terms of autoimmunity, butyrate modulates adaptive immunity, mitigating chronic inflammation by rebalancing anti-inflammatory and pro-inflammatory responses of subpopulations of T and B lymphocytes. However, the role of butyrate in ameliorating chronic inflammation still cannot be fully explained because chronic inflammation in RA involves numerous innate immune cells, such as macrophages and neutrophils. Previous studies demonstrated the limiting effect of butyrate on immune cells such as neutrophils and macrophages in the intestinal mucosa during inflammatory bowel disease (94, 95). Accordingly, the benefits of dietary butyrate for arthritic mice may also involve these innate immune cells. However, this conjecture needs to be confirmed experimentally.

Therapeutically, the butyrate produced from dietary fiber may modulate the immune response in preclinical or clinical RA individuals and reduce pro-inflammatory cytokine expressions (96). Moreover, butyrate also plays a role in maintaining gut microbiota diversity and intestinal barrier function. Butyrate not only promotes the expansion of probiotic genera (such as Lachnospira, Prevotella, and Lactobacillus) but also enhances the function of the intestinal epithelium (97). These therapeutic effects of butyrate are described in detail in this review. Therefore, increasing butyrate concentration through nutrition and diet intake is a promising therapeutic measure for RA. Supportedly, the well-known Mediterranean diet has been demonstrated to be beneficial for body health, gut microbiota diversity, etc. Perhaps the Mediterranean diet should not just limited to people in the Mediterranean region but be more widely encouraged and promoted (98). Additionally, effective extracts from some plants (such as berberine) can not only act on inflammation but also increase the ability of gut microbiota to produce butyrate and improve RA symptoms (99), This finding indicates that increasing the butyrate-producing ability and restoring gut microbiota diversity through drugs may be a promising research direction in the future. However, these therapeutic approaches also face some challenges. Firstly, diet and nutrition intake can achieve therapeutic purposes by increasing the concentration of butyrate (100), but the applicable range is limited. For severe RA, relying solely on diet may not be sufficient to achieve the desired therapeutic effect. Secondly, there are large individual differences in the target population and eating habits; the responses to food and individual gut microbes can vary largely among different individuals. Moreover, changing the original eating habits of patients is also a challenge. There also face challenges in the development of drugs targeting butyrate. As mentioned above, many mechanisms such as the precise regulation of histone modification and ACPA by butyrate are still not clear at present, and long-term studies on RA individuals are still needed to confirm the effect of butyrate on RA prevention and treatment.

To sum up, butyrate locally inhibits intestinal immunity and targets zonulin and TJ proteins to ameliorate intestinal barrier-mediated arthritis. Additionally, butyrate also affects bone metabolism and balances systemic T and B cell immune responses. The effects of butyrate can be summarized into three dominant categories: intestine-joint axis-mediated effects, bone metabolism modulation, and adaptive immunity-mediated immune tolerance. The therapeutic mechanism of butyrate links the gut microbiota and the host. Restoring the gut microbiota’s ability to produce butyrate and supplementing exogenous butyrate are expected to be effective therapeutic methods for RA in the future.
